# Prediction Values for the Influence of Fetal Sex on Plasma Progesterone Concentration in Crioulo Breed Mares: A Preliminary Investigation

**DOI:** 10.1111/rda.70131

**Published:** 2025-10-07

**Authors:** Natália Santana Siqueira de Lara, Romildo Romualdo Weiss, Eunice Oba, Luiz Ernandes Kozicki, Fernando Andrade Souza, Tacia Gomes Bergstein‐Galan, Eloisa Muehlbauer, Mayara Silvestri, Pedro Henrique Lomba de Lima, Eduarda Stankiwich Vaz

**Affiliations:** ^1^ Universidade Federal Do Paraná – Curitiba Paraná Brazil; ^2^ Universidade Estadual Julio Mesquita – Botucatu São Paulo Brazil; ^3^ Pontifícia Universidade Católica – Curitiba Paraná Brazil; ^4^ Universidade Estadual de Ponta Grossa – Ponta Grossa Paraná Brazil

**Keywords:** diagnostic, hormone dosage, sexing

## Abstract

In equine reproduction, determining foetal sex is of economic and strategic importance, but currently available methods are often invasive, costly, or require specialised expertise. This study aimed to assess whether plasma progesterone concentrations could serve as a predictive tool for foetal sex determination in Criollo mares between the 4th and 8th months of gestation and to determine whether these measurements could identify foetal sex. Blood samples were collected at 30‐day intervals from 17 Crioula mares between 114 days and 240 days of gestation. Maternal plasma progesterone concentrations were determined by radioimmunoassay and the sex of the foals was confirmed at birth. Analysis of Variance (ANOVA) was made to verify the variation in maternal progesterone concentrations according to foetal sex and month of gestation. In mares carrying male foetuses (*n* = 7), there was a significant difference in progesterone concentrations between months 4 and 8, as well as between months 7 and 8. In mares carrying male foetuses (*n* = 7), progesterone concentrations were higher (*p* = 0.028) during the 4th month of gestation and lower at the 8th month (*p* = 0.020), compared to the values in mares with female foetuses (*n* = 10). In the 8th month of pregnancy, the sensitivity and specificity of progesterone concentrations for the predicted sex were 80% and 100%, respectively. A limit value of progesterone (12.45 ng/mL) was established through the ROC (receiver operating characteristic) Curve. Prediction values were 78.8% and 100% for males and females, respectively. Detection rates were 100% and 80% for males and females, respectively. The diagnostic accuracy for both sexes was 88.2%. It is concluded that foetal sex influences plasma concentrations of progesterone in pregnant mares.

## 1 Introduction

In human medicine, foetal sexing is commonplace and straightforward, but the use of such tests in veterinary medicine is limited. The value of foetal sexing varies with species and breed. Female offspring are preferred in dairy cattle, whilst higher weight and better feed conversion make males more desirable among beef breeds (Kibushi et al. 2016). In the equine industry, the interest in the sex of foals can vary depending on the breed, activity, and owner preference. In animals used for Polo games, the preference is for females (Pashen et al. 1993; Panarace et al. 2014), whilst in racehorses males are generally preferred (Chezum and Wimmer 1997). Although sexing does not provide control over foetal sex, the information it provides can influence strategic decisions made by the breeder. Foetal sex can directly affect the planning of a particular mating, whether a pregnant mare is sent to auction, and the market or insurance value of the mare (Mcgladdery 2011; Aurich and Schneider 2014).

There are two techniques for determining foetal sex routinely employed in equine reproduction. The first is based on the identification and position of the genital tubercle of the foetus by transrectal ultrasonography, preferably between Days 59 and 68 of gestation (Curran and Ginther 1989). The second technique is based on an assessment of the foetal gonads and external genitalia and can be performed by transrectal ultrasonography (between the 90th and 150th days of gestation) or transabdominal ultrasonography (up to the 220th day of gestation) (Renaudin et al. 1999; Bucca 2005; Livini 2010). Recently, a molecular technique using polymerase chain reaction (PCR) for foetal sexing in the final third of gestation in equines has been developed. This method has high accuracy (95%) and detects free foetal DNA circulating in the maternal blood. Requiring only the collection of blood from the mare, this less invasive method eliminates the inherent risk of rectal examination (De Leon et al. 2012). However, its practical use in the field is limited, and this technique is only employed for research purposes.

Despite the existence of numerous techniques, these tests for foetal sex are rarely used in equine breeding. Current options require expensive equipment and/or examiner experience, limiting their application. The use of this biotechnology could be more widespread if it could be performed with accuracy, in a single evaluation and under field conditions (Mari et al. 2002; Bucca 2005; Aurich and Schneider 2014). An alternative to conventional methods of foetal sexing has been described in elephants; based on plasma progesterone concentrations in pregnant females. In Asian elephants, serum progesterone levels are significantly higher in females carrying male foetuses (210–2280 pg/mL) compared to those carrying females (172–1171 pg/mL), especially between 60 and 65 weeks of gestation (Duer et al. 2007).

To date, there are no studies relating foetal sex to maternal progesterone concentrations in pregnant mares. Thus, our study objectives were evaluation of the influence of foetal sex on plasma progesterone concentrations between the 4th and 8th month of gestation, and verification of the applicability of this test as a technique for foetal sexing.

## 2 Materials and Methods

### 2.1 Animals

The study comprised 17 Crioula breed mares, aged between 5 and 19 years (average 12.8 ± 4.8 years), weighing an average of 420 ± 14.5 kg, housed in a ranch located at latitude 25°32′84″ S and longitude 49°87′84″ W, in the municip of Palmeiras, Paraná. The mares were examined both physically and gynecologically, according to England (2005). All examinations were within normal parameters. After the Transrectal ultrasonographic follicular monitoring of these mares allowed the detection of ovulation, at which time they were covered or inseminated, using Crioulo stallions. From the 4th to the 8th month of gestation, monthly blood samples were collected. The mares were restrained in examination stalls for sample collection by jugular venipuncture. The samples were stored in blood collection tubes with anticoagulant (EDTA), homogenised, and refrigerated at 5°C during transportation.

The collected blood samples were kept at room temperature for 10 min and then centrifuged at 5000 rpm to separate the plasma. Plasma aliquots were placed in identified Eppendorf tubes, frozen, and stored at −20°C until progesterone analysis. The data on progesterone concentrations were categorised according to the gestational month and sex of the foal‐born (Duer et al. 2007). Each sample was categorised according to gestational month as follows: 4 months (Days 114–126—mean 120 ± 3.9 days), 5 months (Days 144–154—mean 149 ± 3.3 days), 6 months (Days 179–188—mean 182 ± 2.7 days), 7 months (Days 209–218—mean 212 ± 2.7 days) and 8 months (Days 237–255—mean 243 ± 3.9 days), with day zero (D0) being the day of ovulation.

### 2.2 Progesterone Concentrations

The samples were analysed at the Laboratory of Animal Reproduction, Universidade Estadual Júlio Mesquita (Unesp, Botucatu/SP), using a commercial radioimmunoassay kit for progesterone determination (Progesterone RIA, Beckman Coulter/Diagnostic Systems Laboratories, CA, USA), following the manufacturer's recommendations. The assay sensitivity was 0.03 ng/mL. The intra‐ and inter‐assay coefficients of variation were below 10% and 15%, respectively, values that are within the acceptable range for hormone assays. This method has been previously validated for equine plasma samples, including pregnant mares (Holtan, Nett, and Estergreen 1975; Holtan, Stabenfeldt, et al. 1975; Legacki et al. 2017).

### 2.3 Statistical Analysis

Data were analysed using a repeated‐measures design to evaluate progesterone concentration across gestational months (4–8) and foetal sex. Normality was assessed using the Shapiro–Wilk test. For mares carrying male foetuses, non‐normal distribution in month 4 led to the application of a non‐parametric Friedman test to detect temporal variation. For mares with female foetuses, repeated‐measures ANOVA was used to assess within‐subject variation. All analyses were conducted using Python (statsmodels and scipy libraries), with significance set at *p* < 0.05. For months in which there was a statistical difference in progesterone concentrations according to foetal sex, threshold values were established. These values were determined using the ROC curve (receiver operating characteristic), prepared by the statistical programme Sigma Plot 12.0 (Systat Software, San Jose, CA). In this way, predictive values for male and female foetuses were defined, using calculations where the number of mares that were carrying foetuses of a certain sex was divided by the number of mares that were predicted to be carrying foetuses of that sex based on plasma progesterone concentrations. The definition of the detection rates of male or female foetuses was given by dividing the number of mares predicted to be carrying foetuses of that sex based on maternal plasma progesterone concentrations by the number of animals of that sex born. Accuracy was calculated by dividing the number of animals that had their sex correctly predicted by maternal plasma progesterone concentrations by the total number of foetuses born.

## 3 Results

Mean plasma progesterone concentrations varied according to foetal sex and gestational month. In mares carrying male foetuses (*n* = 7), a significant variation over time was observed (Friedman test, χ^2^ (4) = 18.4, *p* = 0.001), characterised by a progressive decline from the 4th to the 8th month of gestation. Conversely, mares carrying female foetuses (*n* = 10) exhibited stable progesterone levels across the same period, with no significant variation (repeated‐measures ANOVA, *F*(4, 36) = 0.60, *p* = 0.662). These findings support the existence of a sex‐by‐time interaction in the hormonal dynamics of gestation.

Of the 17 mares evaluated, 10 gave birth to female foals and 7 to male foals. The mean maternal progesterone concentrations by gestational month and foetal sex are presented in Table 1.

**TABLE 1:** Mean and standard deviation of plasma progesterone concentrations (ng/mL) in Criollo mares from the 4th to the 8th month of gestation, according to foetal sex.

Gestational month	Plasma P4 (ng/mL), female gestation	Plasma P4 (ng/mL), male gestation	*p*
4° (*n* = 17)	14.4 ± 7.8a	23.8 ± 14.1bA	0.028
5° (*n* = 17)	10.7 ± 5.7a	17.2 ± 7.7aAB	> 0.05
6° (*n* = 17)	14.1 ± 7.0a	13.3 ± 4.1aAB	> 0.05
7° (*n* = 17)	16.6 ± 4.4a	19.4 ± 5.5aA	> 0.05
8° (*n* = 17)	13.8 ± 6.7a	9.3 ± 1.5bB	0.020

*Note:* Different lowercase and uppercase letters in the same row and column (*p* < 0.05), respectively, by Tukey test. n—number of animals considered in the calculations according to the gestational month; P4—progesterone.

**FIGURE 1:**
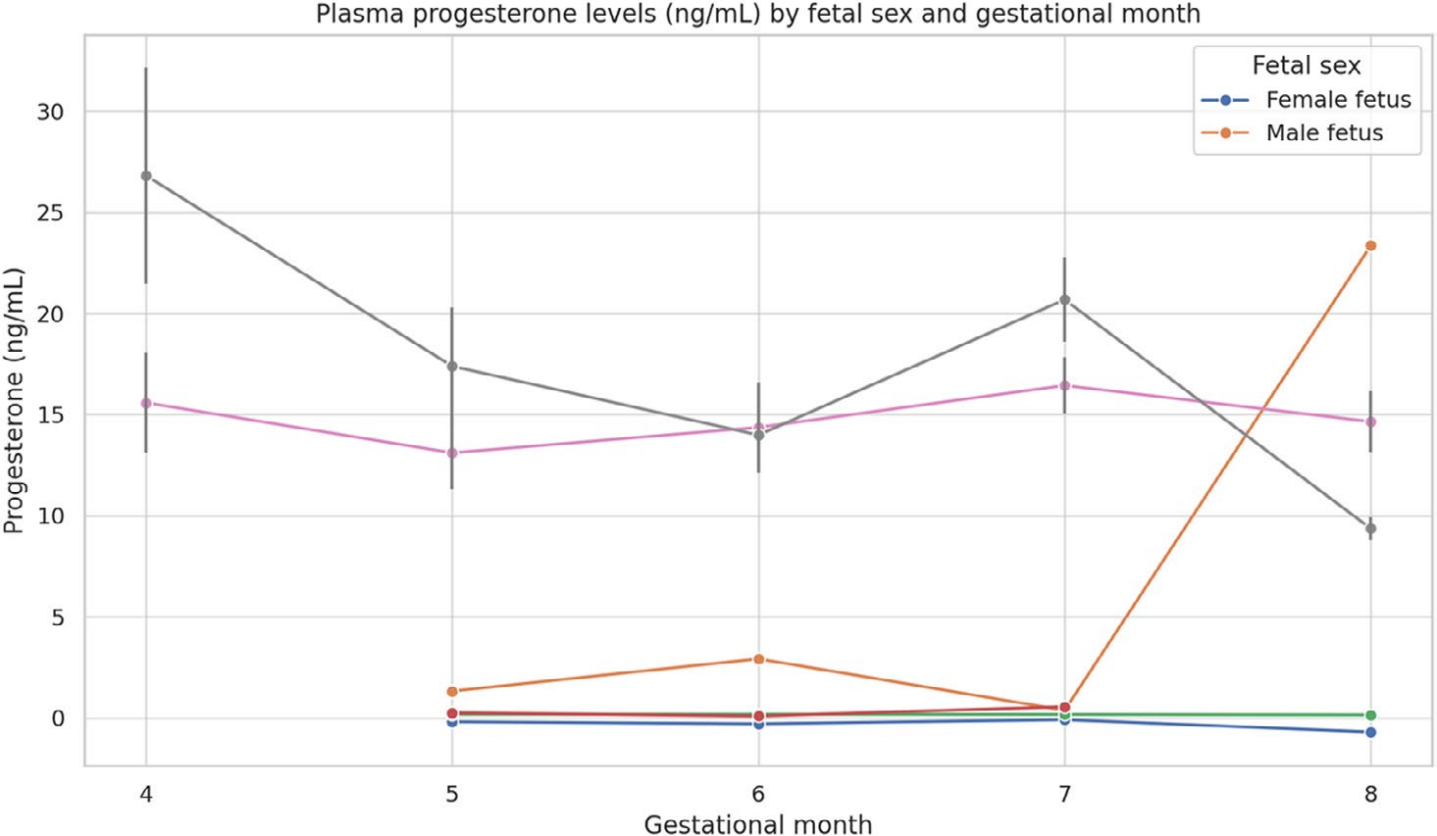
Mean plasma progesterone concentrations (ng/mL) from the 4th to the 8th month of gestation in Criollo mares carrying male or female foetuses. Blue line = female foetuses; orange line = male foetuses; grey line = overall mean of all mares; pink and green lines = standard errors of the mean (SEM) for each group. A significant decrease was observed in mares carrying male foetuses (Friedman test, *p* = 0.001), whereas progesterone levels remained stable in mares with female foetuses (repeated‐measures ANOVA, *p* = 0.662).

When comparing monthly progesterone concentrations, no differences were observed (*p* > 0.05) in mares carrying female foetuses. However, in mares carrying male foetuses, significant differences were detected between months 4 and 8, and between months 7 and 8 (*p* < 0.05), as illustrated in Figure 1.

Progesterone concentrations also differed significantly between mares carrying male and female foetuses in the 4th and 8th months of gestation (*p* < 0.05), with higher values in males at month 4 and in females at month 8. No significant differences were found in months 5, 6, and 7 (*p* > 0.05). Despite the observed difference at month 4, the ROC curve analysis did not yield a meaningful threshold (AUC = 0.17). In contrast, month 8 showed a strong discriminative value (AUC = 0.87), allowing the establishment of a diagnostic cutoff.

**FIGURE 2:**
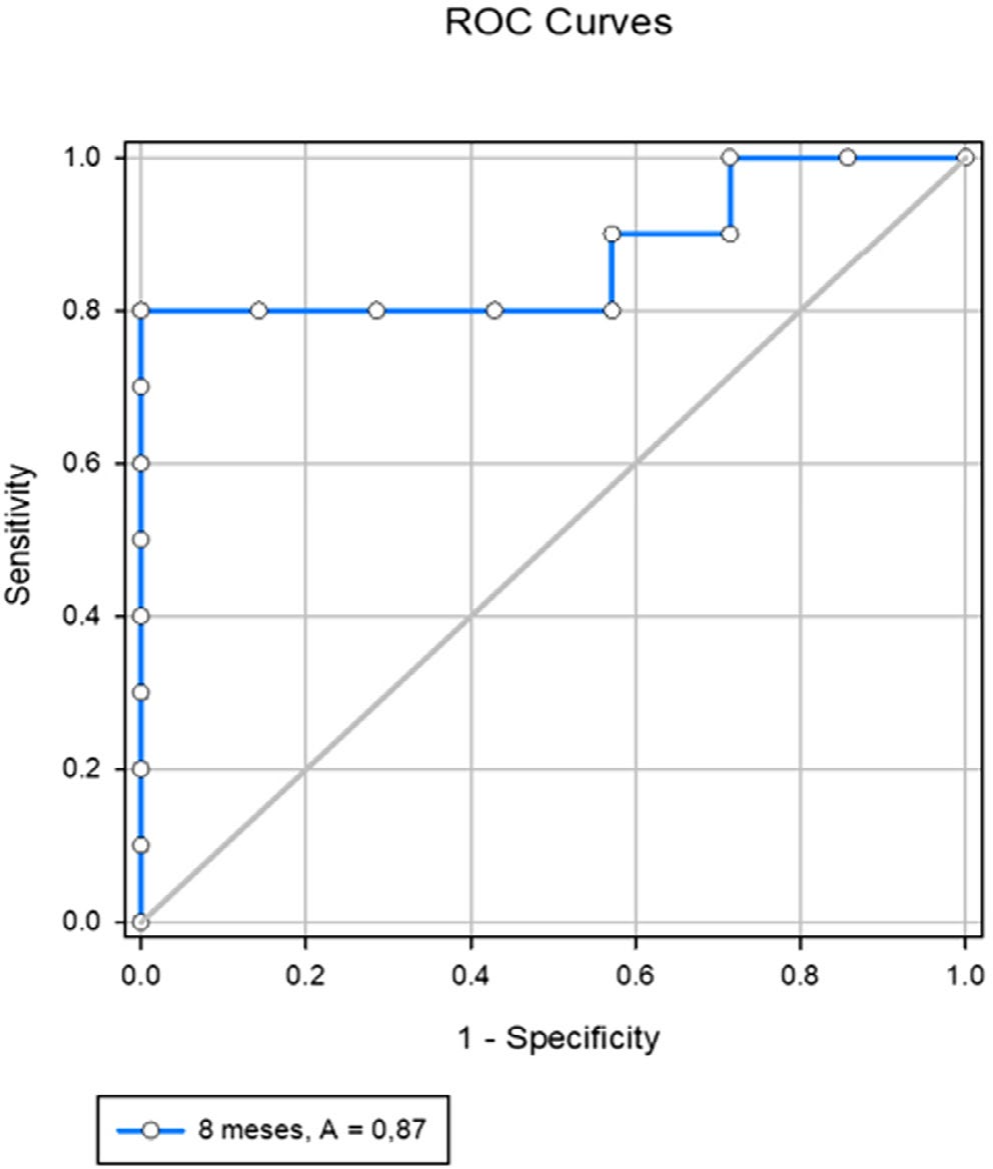
ROC (Receiver Operating Characteristic) curve for maternal plasma progesterone concentration at 8 months of gestation in Criollo mares, indicating the threshold value for foetal sex prediction. AUC = 0.87.

The ROC curve constructed for the 8th month (Figure 2) demonstrated a sensitivity of 80% and specificity of 100%. The threshold progesterone concentration identified was 12.45 ng/mL: mares with plasma progesterone ≥ 12.45 ng/mL were predicted to be carrying female foetuses; values below that threshold indicated male foetuses. The predictive values, detection rates, and overall accuracy are detailed in Table 2.

**TABLE 2:** Predictive values, detection rates, and accuracy of foetal sex prediction based on maternal plasma progesterone concentrations in Criollo mares at the 8th month of gestation.

8th month of pregnancy (*n* = 17)	*n*	Males born	Females born	Predictive values
Mares predicted to be carrying male foetuses by plasma progesterone < 12.45 ng/mL	9	7	2	7/9 (77.8%)
Mares predicted to be carrying female foetuses by plasma progesterone > 12.45 ng/mL	8	0	8	8/8 (100%)
Detection rates		7/7 (100%)	8/10 (80%)	Accuracy 15/17 (88.2%)

## 4 Discussion

No previous equine studies have reported the use of maternal progesterone concentrations to predict foetal sex. Duer et al. (2007) reported the measurement of maternal progesterone concentrations to predict foetal sex in Asian elephants. They showed an increase in serum progesterone concentrations in the first stage of gestation which was greater with male foetuses. Elephants carrying male offspring also had higher mean progesterone concentrations throughout the second phase of gestation. In the third gestational phase, between weeks 60 and 65 the mean serum progesterone concentrations were significantly higher in cows carrying males than females. After 65 weeks there was no significant difference between the sexes.

A refined statistical approach was adopted to explore the temporal dynamics of progesterone concentrations in relation to foetal sex. The analysis revealed a significant decline in progesterone levels over time in mares carrying male foetuses (Friedman test, *p* = 0.001), whilst those carrying female foetuses exhibited stable concentrations throughout the same period (repeated‐measures ANOVA, *p* = 0.662). These findings confirm the existence of a sex‐by‐time interaction in maternal progesterone profiles during mid‐gestation, potentially reflecting differences in fetoplacental signalling or maternal endocrine modulation according to foetal sex, as previously discussed by Clifton (2010), Rosenfeld (2015), and Kalisch‐Smith et al. (2017).

A study of foetal sexing in horses based on plasma testosterone concentrations (Busato et al. 2021) found foetal sex prediction to be more accurate at 8 months of gestation than at 5 months. This corroborates the findings of our study, where predictions were most accurate when based on measurements in month 8. Foetal sex can be determined on ultrasound at 59–68 days and 90–150 days, but this method is not routinely used (Aurich and Schneider 2014; Oliveira et al. 2014). The use of ultrasonography (and its accuracy) is limited by the technique used, the need for high‐quality equipment, the experience of the veterinarian performing the examination, and the mares' tolerance of rectal examination (Curran and Ginther 1989).

In this study, we showed high maternal plasma progesterone concentrations in mares with female foetuses at 8 months. These findings differ from those reported in elephants (Duer et al. 2007), where progesterone concentrations were higher in the final third of gestation in cows carrying male foetuses than those with females. Although significant differences only occurred in the 4th and 8th gestational months, progesterone concentrations initially tended to be higher in mares with male foetuses, and these concentrations decreased until the 8th month, at which point they were significantly lower than the concentrations in mares carrying females. There was an exception to this trend in the 7th month (Figure 2).

Although progesterone concentrations were not measured in the last 3 months of gestation, we speculate that this trend (of decreasing progesterone concentrations in mares) would continue. A study by Holtan, Nett, and Estergreen (1975) showed that progesterone concentrations increased significantly between days 0 and 8 (*p* < 0.05), reaching a maximum on day 64, followed by a slow decline until about day 300, after which they increased in the last 30 days prepartum, falling immediately after parturition and reaching the minimum value on the day after parturition. Progesterone produced by the primary corpus luteum will maintain the pregnancy until around the 40th day when endometrial calyces develop. These calyces produce Equine Chorionic Gonadotropin (eCG) between 33 and 120 days of pregnancy, which has a similar action to FSH, stimulating the growth and luteinization of new follicles, forming accessory corpus luteums that help in the production of progesterone to maintain pregnancy until approximately 150–160 days. After this time, all progesterone is produced from the placenta (Caixeta et al. 2008; Salles and Araújo 2010).

There is little data on equine foetal endocrinology due to the difficulty in collecting foetal blood samples in this species (Ousey 2011) and this makes it hard to speculate on the reasons for the differences in maternal progesterone concentrations depending on foetal sex.

Barnes et al. (1978) showed that progesterone concentrations in the foetal circulation exceeded maternal plasma concentrations but did not correlate this finding with foetal sex. They also showed that, in the last 100 days of gestation, umbilical venous progesterone concentrations were 15–20 ng/mL higher than those in the umbilical artery, whilst in the uterine vein progesterone levels were invariably higher than those in the maternal peripheral circulation. There was no obvious change in foetal plasma progesterone as delivery approached (Barnes et al. 1978). Legacki et al. (2017), showed that foetal progesterone is mainly produced by the adrenal glands, rather than the foetal gonads. Thus, one possible explanation of the difference in hormone concentration between the sexes would be changes in synthesis of this hormone in the adrenal glands, however, this study did not correlate concentration with foetal sex. Another hypothesis is that concentration differences are associated with changes in luteinizing hormone (LH), and in studies by Nakai et al. (2007) and Dhakal et al. (2011), it was observed that female neonates had higher circulating concentrations of LH than males.

In month 4 of gestation there was a significant difference (*p* < 0.05) between the sexes, however, the ROC curve analysis was unable to demonstrate relevant sensitivity and specificity values for foetal sexing.

We showed significantly higher progesterone concentrations in mares carrying female foetuses during the 8th month of pregnancy. From this, a threshold value for this period was defined by ROC curve analysis (Figure 2). At 8 months of gestation, the predictive sensitivity and specificity values for diagnosing male and female foetuses (threshold value 12.45 ng/mL) were 80% and 100%, respectively. In that same period, the predictive values for diagnosis of male and female foetuses were 77.8% (7/9) and 100% (8/8), respectively. The detection rates for males and females were 100% (7/7) and 80% (8/10), respectively. These results are similar to those of Busato et al. (2021), who obtained predictive values for foetal sex diagnosis in mares (based on maternal testosterone concentration) in the 8th month of gestation between 80% and 90%, and detection rates for those authors were between 81.8% and 88.9%.

At 8 months of gestation, the accuracy of maternal plasma progesterone concentration for predicting foetal sex was 88.2% (15/17) (Table 2) in this study. In conventional methods of foetal sexing in horses, the accuracy is usually higher, from 65% to 97.6% in sexing through the identification of the genital tubercle (Curran and Ginther 1989, 1993; Curran 1992; Merkt et al. 1999; Mari et al. 2002; Holder 2003; Taveiros et al. 2008; Livini 2010), and can reach up to 99% accuracy through the identification of foetal gonads and external genitalia (Holder 2003), and in sex determination through the detection of circulating free foetal DNA, the accuracy was 95% (De Leon et al. 2012). In the study by Busato et al. (2021), using maternal plasma testosterone concentrations for the 8th month of gestation, the accuracy was 85%.

Maternal plasma progesterone concentrations are a viable alternative for the determination of foetal sex in more advanced pregnancies. The advantages of this method are that it is a less invasive test, with reduced morbidity for the mare, and allows its use in small breed mares. In addition, this test can be used to predict foetal sex at a gestational age that has not been studied for other techniques.

## 5 Conclusions

Foetal sex influences maternal plasma progesterone concentrations. Foetal sex prediction based on maternal plasma progesterone concentrations can be performed in the 8th month of gestation.

## Author Contributions

N.S.S.L. conceived and conducted the experiment and drafted the manuscript. R.R.W. supervised the study. E.O. performed the radioimmunoassay laboratory analyses. L.E.K. contributed to the co‐supervision and statistical analyses. F.A.S. and T.G.B.‐G. contributed to co‐supervision and manuscript review. E.M. performed statistical analyses. M.S. and E.S.V. contributed to manuscript writing. P.H.L.L. revised the statistical analyses. All authors read and approved the final version of the manuscript.

## Ethics Statement

The study was approved by the Ethics Committee on Animal Use of the Agricultural Sciences Sector of the Federal University of Paraná (protocol number 030/2020).

## Conflicts of Interest

The authors declare no conflicts of interest.

## Data Availability

The data that support the findings of this study are available from the corresponding author upon reasonable request.
